# Genetic diversity of *Plasmodium falciparum* histidine-rich protein 2 (*Pf*HRP2) and its effect on the performance of *Pf*HRP2-based rapid diagnostic tests

**DOI:** 10.1186/s13104-019-4361-6

**Published:** 2019-06-11

**Authors:** Ali Mussa, Mustafa Talib, Zeehaida Mohamed, Khalid Hajissa

**Affiliations:** 1 Genetics and Molecular Biology Laboratory, Department of Zoology, Faculty of Science, University of Khartoum, Khartoum, Sudan; 2 Department of Zoology, Faculty of Science and Technology, Omdurman Islamic University, B.O.Box382, Omdurman, Sudan; 3 Department of Medical Microbiology & Parasitology, School of Medical Sciences, Universiti Sains Malaysia, 16150 Kubang Kerian, Kelantan Malaysia

**Keywords:** RDTs, Histidine-rich protein, Polymorphism, *Plasmodium falciparum*, Omdurman, Sudan

## Abstract

**Objective:**

Rapid diagnostic tests (RDTs) play a crucial role in the management and control of malaria infection. The histidine-rich protein 2 (*Pf*HRP-2) based RDTs are the most commonly used RDTs for malaria diagnosis in Sudan. Deletion of *pfhrp*2 in *Plasmodium falciparum* genome affect the accuracy of *Pf*HRP-2 based RDT kits. This study aimed to identify molecular variation of *pfhrp2* among suspected malaria patients from different clinics in Omdurman, Sudan.

**Results:**

A noticeable variation between the RDT (Alltest Biotech, China) and nPCR results was observed, for RDT 78% (46/59) were *P. falciparum* positive, 6.8% (4/59) were co-infected with both *P. falciparum* and *Plasmodium vivax*, 15.3% (9/59) were negative by the RDT. However, when the nPCR was applied only 44.1% (26/59) and 55.9% (33/59) was *P. falciparum* positive and negative respectively. The *pfhrp2* was further amplified form all nPCR positive samples. Only 17 DNA samples were positive from the 26 positive *P. falciparum*, interestingly, variation in band sizes was observed and further confirmed by DNA sequencing, and sequencing analysis revealed a high-level of genetic diversity of the *pf*hrp2 gene in the parasite population from the study area. However, despite extreme sequence variation, diversity of *Pf*HRP2 does not appear to affect RDT performance.

## Introduction

Despite the substantial efforts to control or eliminate the disease, malaria remains the leading cause of morbidity and mortality in tropical and sub-tropical countries including Sudan [1]. About 87% of the population is in high malaria transmission areas. Generally; 5–10% of malaria infections in Sudan caused by *P. vivax* and the remaining infection is caused by *P. falciparum* [1, 2]. The early and accurate diagnosis is crucial for the disease control and management. Moreover, early instituted treatment remarkably improved the clinical outcomes. Rapid diagnostic tests (RDTs), play a key role in malaria initial diagnosis as well as treatment monitoring [3]. RDTs permit a reliable malaria detection particularly in remote areas that lacks good quality microscopy services or PCR assays [4]. Therefore, it becomes the focal point of malaria control in many parts of the world. The availability along with the scale of use of this tests have increased dramatically in the recent years [5]. The most currently in use RDTs target histidine-rich protein 2 (HRP2) specifically presents in *P. falciparum* and/or lactate dehydrogenase (pLDH) or aldolase that can detect all the *Plasmodium* species [6].

*Pfhrp2* is single copy subtelomeric gene located on chromosome 8 that code for histidine-rich protein 2 (HRP2). The amino acid sequences of the protein are formed in great quantity on the surface of infected RBCs during the asexual and early gametocyte stages of *P. falciparum* infection [3, 7–10].

Histidine-rich protein 2-based RDTs performance can be influenced by several factors such as protein antigenic diversity, HRP2 persistence in the circulation following parasites clearance, and the density of the parasite [5].

A key apprehension is the genetic variation in the *Pf*HRP2 amino acid sequences between parasites isolated from geographically different places, which may cause false-negative results in rapid diagnostic tests (RDTs). Accordingly, this study was aimed to investigate the possible variations in *Pfhrp2* gene which might influence the accuracy of the diagnostic test in the study area.

## Main text

### Methodology

#### Study population

The study population was suspected individuals with *P. falciparum* infection from different Clinical Centers in Omdurman city. A total of 59 suspected patients were enrolled in this study, 27 were females and 32 males. About 3 ml of peripheral blood was collected in EDTA vacutainer tubes for biochemical analysis, as well as for *Plasmodium* species identification (RDT), and then stored at 4 °C for DNA extraction.

#### Detection of malaria infection by ICT

For parasite detection, Test™ Malaria *P. f*/*P. v* rapid test cassette (whole blood) was used according to the manufacture instruction. Briefly, 5 μl l of whole blood was added to the sample bad. The blood was then reacted with the dye conjugate, and the mixture migrated upward on the membrane, reacted with anti-HRP2 antibodies on the *P. f.* test line region and with anti-pLDH antibodies on the *P. v*. test line region. The test was considered positive if a colored line was appeared in *P. f.* line region or *P. v.* line region or both.

#### DNA extraction

A total of 200 μl of whole blood was used for the isolation of the genomic DNA by using G-DEX™IIb Genomic DNA Extraction Kit (iNtRON, South Korea) and the extracted DNA was kept in − 20 until further use.

#### Parasite identification and amplification of pfhrp2 by polymerase chain reaction (PCR)

*Plasmodium* genus and species-specific nested PCR were performed as a two stage procedures as described by Snounou et al. [11]. Subsequently, the *pfhrp2* gene was amplified by PCR using Pfhrp2-F1 (5′-ATTATTACACGAAACTCAAGCAC-3′) and Pfhrp2 R1 (5′-AATAAATTTAATGGCGTAGGCA-3′) primers. The PCR reaction was performed in final volume of 20 µl containing 4 μl of 5× HOT FIREPol Blend Master Mix, 1.5 μl (0.75 μM) form each primer, 2 μl (100 ng/μl) of and sterile ddH_2_O were added to make the final volume of 20 µl. The PCR amplification was carried out under the following conditions: initial denaturation 95 °C for 4 min, followed by 35 cycles of amplification at 95 °C for 30 s, 58 °C for 30 s, and a final extension cycle a 72 °C for 1 min.

#### DNA sequencing

Selected *Pf*hrp2 positive samples were sent for DNA sequencing to Macrogen Laboratory Company. The results were analyzed using BioEdit Sequence analysis software (Ibis Therapeutics, USA). The sequence data were analyzed using the Basic Local Alignment Search Tool (BLAST) through the NCBI website. Nucleotide sequences were translated to amino acid sequences using ExPASy translate tool. New amino acid repeat sequences were identified in addition to the previously recognized repeats and new numeric codes were given [5].

#### Statistical analysis

The Statistical Package for Social Sciences (SPSS, IBM, and Chicago, USA Version 23.0.3) was used for data entry and data analysis. All the data were double checked and prepared properly whilst being documented in order to detect missing data or errors.

### Results

**Fig. 1:**
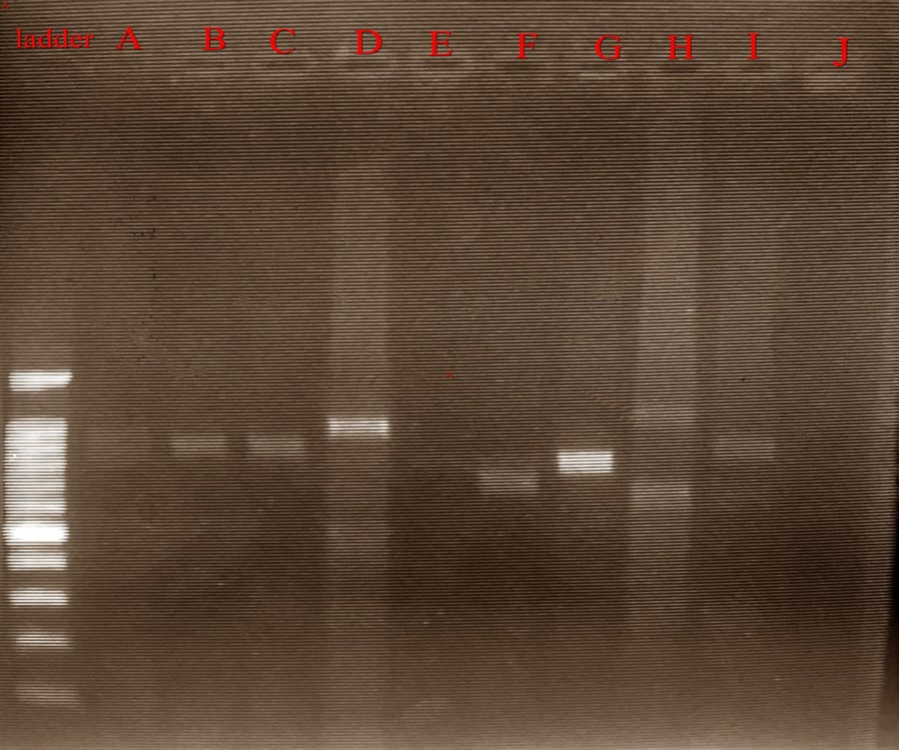
Amplification of *Pf*hrp2 gene, lane ladder: 100 bp DNA marker, lane A–J: PCR amplicons (~ 791,800, 1 Kbp) of amplification for *Pfhrp2* gene from different samples

The ICT results showed that 46 samples out of 59 were positive for *P. falciparum* with percentage of 78%. While 4 samples were co-infected with *P. falciparum* and *P. vivax* with percentage of 6.8%, and 9 (15.3%) samples were negative. On the other hands only 26 (44.1%) were positive by nPCR (detecting of ~ 205 PCR amplicon) and 33 (55.9%) were negative. In addition, Out of 26 samples, only 17 samples were positive for *Pfhrp2* gene. Whereas 9 samples were negative for the same gene. Among the 17 positive samples, 16 showed variations and their length was not corresponding to the reference lengths which retrieved from the gene-bank database, and one sample showed no variation as it is length was corresponding to the reference as shown in Fig. 1.

#### DNA sequence analysis

##### Variation in PfHRP2

PCR-amplified *pfhrp2* fragments varied in band size from 700 to 1000 bp. Most of this variation resulted from the presence of many number of repeats (27-, 18-, and 9-bp). When translated into amino acid sequences, *Pf*HRP2 consisted of varying numbers of 13-, 11-, 9-, 6-, and 3-aa repeats. Furthermore a total of 16 different amino acid repeats were identified from the *Pf*HRP2 sequences of three samples.

##### Repeat types and frequency variation of Pfhrp2

The analysis of the randomly sequenced three samples revealed that corresponding amino acid sequences contain type 25, 2 and 1 motifs in the N terminal respectively. While, in C terminal of both two samples contain type 12 motif and the third have type 26 motif. In particular, type 2 was repeated 36 times, type 4 motif was repeated 16 times, type 6 motif was repeated 14 times, type 7 motif was repeated 9 times, and type 9 was repeated six types. Type 10 and 14 motifs were repeated 3, 2 times. In comparison, the less frequent motif was type 1 repeated 1 time. Type 3, type 5, type 8, type 11 and type 13 repeats were not found in this study.

Seven new types of repeat motifs were identified in the present study and were given new numeric codes (Table 1). The total number and the number of each repeat varied within and between sites. Types and frequency were: type 25 and type 26, type 27, 31 were repeated 1 time for each respectively, type 28 was repeated 3 times, type 29 was repeated 2 times, type 30 was repeated 8 times.

**Table 1:** Amino acid sequences of *Pf*HRP2 repeats

Name of the repeat	Amino acid repeat sequence	Antigen observed (*Pf*HRP2)
Type 1 motif	AHHAHHVAD	+
Type 2 motif	AHHAHHAAD	+
Type 3 motif	AHHAHHAAY	−
Type 4 motif	AHH	+
Type 5 motif	AHHAHHASD	−
Type 6 motif	AHHATD	+
Type 7 motif	AHHAAD	+
Type 8 motif	AHHAAY	−
Type 9 motif	AAY	+
Type 10 motif	AHHAAAHHATD	+
Type 11motf	AHN	−
Type 12 motif	AHHAAAHHEAATH	+
Type 13 motif	AHHASD	−
Type 14 motif	AHHAHHATD	+
Type 25 motif	AHHVAD*	+
Type 26 motif	AAA*	+
Type 27 motif	AHHAHHVDT*	+
Type 28 motif	AHHAHHAPD*	+
Type 29 motif	AHHAPH*	+
Type 30 motif	AHHAPD*	+
Type 31 motif	AHHAHHASN*	+

(+): motif present, (−): motifs absent, (*): repeat types not previously reported

### Discussion

Malaria RDTs represent prodigious potential for quick and accurate diagnosis of malaria infections, which could lead to prompt and appropriate management and control of the disease [3]. Obtaining malaria RDTs with high sensitivity and specificity is crucial for using of these devices. Unfortunately, the genetic diversity within the *Pf*HRP2 antigens used in the RDTs has the possibility to influence their sensitivity [3]. Knowing the population structure of this gene will allow better designing of this kind of test and reduce the false negative results [12]. Therefore, this study was conducted to pinpoint the prevalence of *Pfhrp2* gene variations in *P. falciparum* parasites among suspected malaria patients attending different clinics in Omdurman-Sudan.

A total of 59 malaria suspected individuals blood samples were screened by ICT, based on this screening, 46 samples were positive for *P. falciparum,* 4 were co-infected with *P. falciparum* and *P. vivax* and 9 were negative. In addition, nPCR confirmed 26 samples were positive for *P. falciparum* including 6 samples from the 9 negative and 1 sample from the 4 co-infection while they misdiagnosed by ICT.

Amplification of 26 *P. falciparum* samples for *Pfhrp2* gene showed that 17 were positive and 9 samples were negative for the gene. On the other hand, out of 1392 samples, 1391 samples were amplified for *Pfhrp2* and one sample was negative in study conducted in India [8]. Moreover from 97 *P. falciparum* isolates, amplification of *Pfhrp2* was successful in 93 isolates and 4 samples failed to amplify any *Pfhrp2* fragments in China–Myanmar border [13].

From the 17 positive 16 samples were shown variation comparing to the reference sample, whereas 1 sample nearly close to the reference sample which might indicate no variation based on the gel image, figure (5), similar to study conducted in Senegal [7].

Moreover, the *Pf*HRP2 protein sequences from exon 2 are more complicated and contain varying numbers of different types of amino acid repeats [12]. Representative samples were sent for sequencing and the results indicated that the variation of band size is due to the presence of different numbers of 27-, 18-, and 9-bp repeats samples as same as in the study conducted in India [8]. When this repeats translated into amino acid sequences, *Pf*HRP2 consisted of varying numbers of 13-, 11-, 9-, 6-, and 3-aa repeats representing similarity with Baker et al. [12]. Furthermore, 16 different repeats were reported in the present study similar as in study conducted in India [8] whereas in China–Myanmar border area 33 repeat variants were reported [13], however in other study conducted in Australia only 14 repeats variants were identified even though samples were collected from 19 different countries [12] and again 20 repeat variants were also detected in Africa, South and Central America [3]. The findings suggest that sequence diversity in *Pfhrp2* gene in study area is not likely to negatively influence performance of currently used *Pf*HRP2 RDT, as same as in study performed by taking 458 isolates of *P. falciparum* collected from 38 countries [12]. In contradiction with study conducted in Peru that diversity in *Pfhrp2* gene has positive effect in the performance of *Pf*HRP2 RDT [14].

On the other hand, the sequence of the samples began in the N terminal with type 25 motif, type 2 motif and type 1 motif respectively, similarly, two samples ended in the C terminal with a type 12 motif and the third sample ended with a type 26 motif. Whereas in other studies the sequences start with type 1 motif in the N terminal and end in C terminal with type 12 motif, moreover, the frequency of each repeat type in the current study showed marked variation with similar global studies [7, 12, 13].

### Conclusion

In summary, sequence analysis has identified new repeats in addition to the reported identified in previous studies which increase the genetic diversity of the *pfhrp2* gene, additional reason for genetic variation was reflected by the presence of different types of repeats, as well as variations in sequence, copy number, and arrangement of these repeats. However, no impact of this great variation noticed in this gene in the performance of RDT in the study area. Moreover, the data obtained in this study could be the baseline for future studies and may lead to a better understanding of the *Pfhrp2* gene structure and how its variation contributes to the RDT positivity and in turn, also help in the generation of improved malaria RDTs.

## Limitations

Even though this study showed that diversity of *Pf*HRP2 does not appear to affect RDT performance, despite extreme sequence variation, the study was limited by the small sample size and also RDT negative samples was not included. However, the study still ongoing to enroll more samples.

## Data Availability

All original or analyzed data for this study is available on request from the corresponding author.

## Abbreviations

nPCR: nested polymerase chain reaction
*P. f*: *Plasmodium falciparum*
*P. v*: *Plasmodium vivax*
BLAST: Basic Local Alignment Search Tool
ICT: immune chromatographic test
bp: base pair
aa: amino acid
RBCs: red blood cells
HRP-2: histidine-rich protein 2
RDTs: rapid diagnostic tests
*Pf*HRP-2: *Plasmodium falciparum* histidine-rich protein 2
pLDH: lactate dehydrogenase
